# Exhaled aldehydes as promising compounds to describe the energy balance of lactating dairy cows on a fresh herbage–based diet

**DOI:** 10.3168/jdsc.2025-0809

**Published:** 2025-11-21

**Authors:** J. Eichinger, A.-M. Reiche, L. Eggerschwiler, M. Tretola, L. Pinotti, L.K. Tintrop, P. Fuchsmann, K. Huber, F. Dohme-Meier

**Affiliations:** 1 Ruminant Nutrition and Emissions, Agroscope, 1725 Posieux, Switzerland; 2 University of Hohenheim, Institute of Animal Science, 70599 Stuttgart, Germany; 3 Research Contracts Animals, Agroscope, 1725 Posieux, Switzerland; 4 Swine Research Group, Agroscope, 1725 Posieux, Switzerland; 5 Department of Veterinary Medicine and Animal Science, University of Milan, 26900 Lodi, Italy; 6 Human nutrition, sensory analysis and flavour, Agroscope, 3097 Bern, Switzerland

## Abstract

The energy balance (EB) of dairy cows is typically assessed through calculated metrics of energy intake and expenditure, which require elaborate individual measurements. Increased concentrations of biomarkers, such as BHB and nonesterified fatty acids (NEFA), are associated with a catabolic status and require individual measurements through invasive blood sampling for reliable and precise assessment. Exhaled volatile organic compounds (VOC) could be a promising low-invasive alternative, but their suitability for this purpose has been scarcely studied. Therefore, the aim of this study was to identify exhaled VOC correlated with the EB of dairy cows and compare their association with that between EB and serum BHB and NEFA concentrations. For this purpose, we used data from a feeding trial including 34 early-lactating dairy cows that were fed freshly cut herbage and concentrates according to their requirements for 6 wk. Exhaled breath and blood were sampled on 2 consecutive days (same time of the day) in lactation wk 5, 7, and 10, and EB was calculated per lactation week. Exhaled breath was subjected to untargeted VOC analysis, and serum was analyzed for BHB and NEFA concentrations. To identify exhaled VOC associated with EB, we selected 19 cows that exhibited calculated negative EB (NEB) in lactation wk 5 (average DIM 34.6 ± 13.4) and positive EB (PEB) in lactation wk 10 (average DIM 76.6 ± 13.4). Using this subset of cows, we then performed partial least squares-discriminant analysis (VOC data) and Wilcoxon signed rank tests (VOC data and serum BHB and NEFA concentrations) to investigate the differences between cows that initially exhibited an NEB followed by a PEB. Three exhaled VOC appeared to be discriminatory and differed between NEB and PEB of cows: the fatty aldehydes octanal, nonanal, and decanal. Serum BHB and NEFA concentrations were higher in cows exhibiting NEB than in cows with PEB. Using the data of all cows across all lactation weeks, we performed repeated measures correlations between EB, discriminatory VOC, and serum BHB and NEFA. The EB correlated positively with relative concentrations of octanal, nonanal, decanal, serum BHB, and serum NEFA. Relative concentrations of exhaled decanal, but not octanal and nonanal, correlated with serum BHB and NEFA concentrations, respectively. These findings suggest an association between exhaled fatty aldehydes and the energy status of cows that is as strong as that of serum BHB and NEFA. The physiological role and potential of these metabolites as new biomarkers of NEB in dairy cows and the causality of the association should be further investigated.

## Highlights

• Exhaled VOC were linked to the EB of cows fed fresh herbage.
• Correlations of EB with serum BHB and exhaled fatty aldehydes were comparable.
• Exhaled fatty aldehydes may be promising biomarkers for energy status in cows.

## Graphical Abstract

**Figure:**
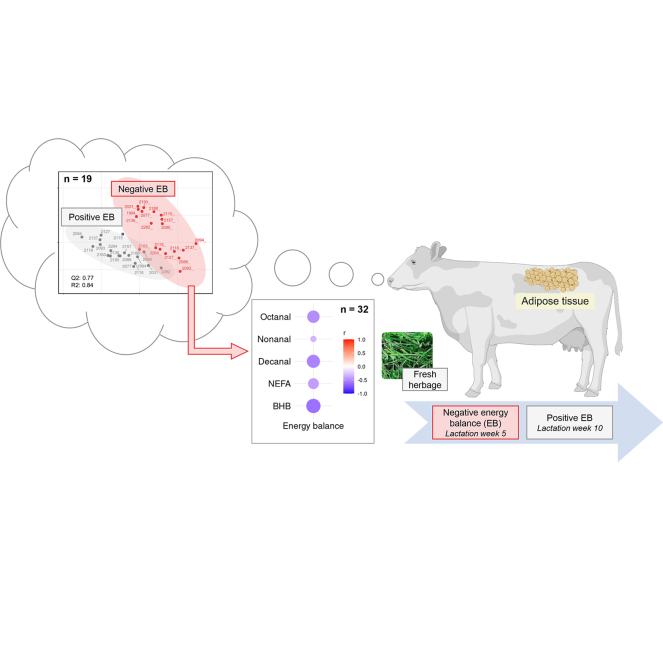
**Summary:** We investigated exhaled breath to explore potential new biomarkers for the energy balance (EB) of dairy cows fed fresh herbage. Cows (n = 19) exhibiting negative (lactation week 5) and positive (lactation week 10) EB differed in their exhaled-breath profiles, namely in certain exhaled volatile organic compounds (VOC). The most discriminating exhaled VOC were the fatty aldehydes octanal, nonanal, and decanal. These VOC and serum β-hydroxybutyrate (BHB) and nonesterified fatty acids (NEFA) correlated with the calculated EB in 32 cows. Thus, exhaled fatty aldehydes could be considered in assessing the energy status of dairy cows.

In early-lactating, high-yielding dairy cows, energy intake often does not meet the requirements for both maintenance and milk production, resulting in a negative energy balance (**NEB**) and mobilization of body reserves such as adipose tissue (van Erp-van der Kooij et al., 2023). Negative energy balance can be associated with subclinical and clinical ketosis, reduced animal welfare, and decreased performance (Suthar et al., 2013). Therefore, it is crucial to assess the energy status of dairy cows to inform the adjustment of feeding and management when necessary. A cow's energy balance (**EB**) can be assessed through calculations. However, BW and individual feed intake, which are necessary for the calculation, are rarely measured on farms. Therefore, EB is rarely monitored in the field. Possible NEB-related catabolic states are commonly assessed by invasive blood sampling, with high BHB and nonesterified fatty acid (**NEFA**) concentrations indicating the mobilization of body fat due to low energy intake (Newman and Verdin, 2014). Milk characteristics, such as milk fat-to-protein ratio or urinary ketone concentrations, provide an alternative but are limited in their precision and reliability because of significant interanimal variation; therefore, difficulties arise in setting general thresholds (Friggens et al., 2007; Serrenho et al., 2022). Exhaled VOC (compounds from C6 to C16) could be a promising low-invasive alternative (van Erp-van der Kooij et al., 2023). The very VOC (**VVOC**; compounds with <C6) acetone changes in response to ketosis and is correlated with serum BHB and NEFA as well as with milk acetoacetate and acetone concentrations (van Erp-van der Kooij et al., 2023). Other than acetone, larger compounds, including VOC, have been so far less explored, but they could be additional, sensitive markers for early detection of NEB. In particular, it remains unclear whether VOC could possibly be markers for EB in nonketotic situations and, if so, how strongly they are associated in comparison to serum BHB and NEFA concentrations.

Therefore, the aim of this study was to evaluate the suitability of exhaled VOC to discriminate between the metabolic conditions of NEB and positive EB (**PEB**) in individual cows transitioning from NEB to PEB. Furthermore, the strength of the correlations between the calculated EB and exhaled VOC and those between EB and serum BHB and NEFA were used to assess the validity of this VOC-based discrimination of metabolic conditions, such as NEB and PEB. The data used originated from a feeding trial involving cows in early lactation.

The study was conducted at Agroscope (Posieux, Switzerland) between April and October 2022. The experimental protocol complied with Swiss legislation for animal welfare and was approved by the Animal Care Committee of Fribourg Canton, Fribourg, Switzerland (2021–38-FR). The study was part of a feeding experiment comparing 3 concentrate types for early-lactating dairy cows for 6 wk each in the spring and autumn of 2022 (Reiche et al., 2025). Thirty-four primiparous (n = 4) and multiparous (n = 30) early-lactating Holstein cows were involved in this study, with 17 cows in the spring run and 17 cows in the autumn run. At the start of each run, the average DIM ± SD was 33.2 ± 15.1, and the average milk yield ± SD was 37.8 ± 7.4. The sample size (n = 34) constituted a convenience cohort and was originally determined for the experiment by Reiche et al. (2025) through power analysis for linear mixed-effects models, using reticular pH as the primary outcome variable. All animals available from that experiment were included in the present study. In both runs, the cows were housed in a freestall barn equipped with access-controlled feed-weight troughs (Insentec RIC, Hokofarm Group, Marknesse, the Netherlands) and an automatic concentrate feeding station (Insentec RIC, Hokofarm Group, Marknesse, the Netherlands). They were fed an ad libitum freshly cut herbage supplemented by concentrate feed. Balanced for milk yield, lactation number, and DIM, the cows were assigned to 3 concentrate types similar in protein and energy content (as-fed basis): a cereal-based control concentrate (**CON**, n = 11; corn, 31%; barley, 31%; wheat, 33%; corn gluten, 3%; and minerals, 2%), an experimental concentrate (**EXP1**; n = 12; bakery byproducts, 55%; corn, 31%; corn gluten, 5%; straw meal, 5%; and minerals, 3%), or an experimental concentrate in which the straw meal was replaced by cocoa bean shells (**EXP2**, n = 11). The concentrates were offered independently of milk production at the automatic feeding station, starting with 5 kg of concentrates per day in lactation wk 5 and gradually increasing in line with increasing milk yield to 6 kg per day from lactation wk 7 onward. All cows had free access to fresh water.

Feed samples were collected and analyzed as described by Reiche et al. (2025). Throughout the experiment, individual feed intake was continuously recorded. Milk yield was recorded automatically twice daily (Fullwood, A. Bertschy AG, Guschelmuth, Switzerland), and milk samples for the analysis of milk composition were collected twice weekly in the evening and in the subsequent morning milking. These 2 milk samples were pooled according to the individual evening and morning milk yields to produce a 50-mL aliquot preserved with bronopol for further analysis of fat, protein, and lactose contents, using a Milkoscan FT6000 (Foss Electric, Hiller⊘d, Denmark). The BW of each cow was recorded twice daily after milking using a walkover weighing system (Insentec/Hokofarm Group BV, Marknesse, Netherlands). Maintenance requirements and milk energy output were determined according to Agroscope (2021). The EB (MJ NEL) was calculated per cow and lactation week, as described by Gross et al. (2011).

The collection of exhaled breath and blood took place at 3 time points, which corresponded, on average, to lactation wk 5 (**W5**), 7 (**W7**), and 10 (**W10**). Sampling was initiated in W5 to target cows in a relevant NEB phase while simultaneously avoiding early postpartum inflammatory processes that occur after parturition (Huber, 2024) and may confound exhaled-breath profiles. Exhaled breath was collected on one day, and blood was collected on the following consecutive days, both within the same timeframe after morning milking (from 0600 h to 0800 h) and before providing fresh feed, to exclude any potential effects of circadian rhythm and feed ingestion. Exhaled breath and blood were analyzed for exhaled VOC and serum BHB and NEFA concentrations, respectively. The exhaled-breath sampling approach was based on solid phase extraction (**SPE**) cartridges containing a highly porous polystyrene-divinylbenzene copolymer HR-P, 50–100 μm, 3 mL/200 mg (Macherey-Nagel, Oensingen, Switzerland), which were conditioned using nanopure water, methanol, acetone, and acetonitrile, as described in detail by Eichinger et al. (2024) and Eichinger et al. (2025b). The conditioned SPE cartridges (duplicates for each cow) were connected to the technical setup for sampling exhaled VOC for 3 min, following the method of Eichinger et al. (2024). After sample collection, the SPE cartridges were stored at 4°C until preparation for VOC analysis. Blood samples from the jugular vein were collected into a 9-mL tube containing a clot activator (Vacuette, Greiner Bio-One GmbH, Kremsmünster, Austria) by venipuncture and were stored for 1 h at room temperature until centrifugation at 3,000 × *g* for 15 min and then at 4,000 × *g* for 2 min at 4°C each. The supernatant serum was transferred into 1.5-mL tubes and stored at –20°C until analysis of the BHB and NEFA concentrations (Randox Laboratories Ltd., Crumlin, UK). Exhaled-breath samples were prepared and analyzed as described by Eichinger et al. (2024). For exhaled VOC data, MS signals were deconvoluted according to Eichinger et al. (2024), and manual peak integration was performed using MassHunter Quantitative Analysis software (version 12.1; Agilent Technologies, Santa Clara, CA). The data were normalized using probabilistic quotient normalization (Dieterle et al., 2006). The exhaled VOC concentrations reported in the text refer to relative concentrations determined from the peak area of the VOC (arbitrary unit).

For data analysis, data from 2 sick cows were excluded. To assess the potential confounding effects of concentrate type and season (spring vs. autumn) and to determine the need for their inclusion in the statistical analyses, we evaluated the effects of the concentrate type and season on the studied variables using linear models (Reiche et al., 2025) and partial least squares-discriminant analysis (**PLS-DA**; MetaboAnalyst; version 6.0; Xia et al., 2009). Concentrate type and season did not affect BHB and NEFA concentrations, except for NEFA concentrations in autumn, which were lowest in CON cows, intermediate in EXP1 cows, and greatest in EXP2 cows (Reiche et al., 2025). Similarly, concentrate type and season did not influence EB (data not presented) or VOC profiles because no valid PLS-DA model was found (data not presented). Therefore, we proceeded with the main statistical analyses of this work, namely PLS-DA and repeated correlation analysis. As a first statistical analysis, PLS-DA was performed to discriminate between the exhaled VOC profiles of cows that initially exhibited an NEB in W5 and later a PEB in W10. The PLS-DA included the data of W5 and W10 from 19 cows from all 3 concentrate groups and seasons (4 CON, 8 EXP1, 7 EXP2) that exhibited NEB in W5 (EB range: −84.1 to −7.1 MJ NEL) and later PEB in W10 (EB range: 5.43 to 27.3 MJ NEL). To consider any potential influence of concentrate type and season, PLS-DA was also run separately using subsets of the cows of each concentrate type and each season. The PLS-DA models were considered valid when presenting a predictive ability parameter (**Q2**) > 0.5 and a goodness-of-fit value (**R2**) > 0.8 (Wold et al., 2001). For each valid PLS-DA, the variable importance in projection (**VIP**) scores were calculated. Additionally, a Wilcoxon signed rank test adjusted for multiple testing using false discovery rate (**FDR**) correction by the Benjamini–Hochberg procedure (MetaboAnalyst; version 6.0; Xia et al., 2009) was conducted between cows exhibiting NEB and later PEB for each exhaled VOC and for serum BHB and NEFA concentrations. The VOC presenting a VIP score of >2 in all PLS-DA models and a significant Wilcoxon's test after correction (FDR <0.05) were retrieved and used in a second statistical analysis for further repeated measures correlation analysis. The latter was performed using data from all 32 healthy cows in all 3 lactation weeks studied and assessed correlations between serum BHB and NEFA concentrations and the discriminatory exhaled VOC and EB and correlations between exhaled VOC and serum BHB and NEFA concentrations in R (version 4.3.3; R Core Team, 2023) using the rmcorr package in R (Bakdash and Marusich, 2017). Differences in the strength of correlations were evaluated using the Williams test (Enderlein, 1961) and the cocor package in R (Diedenhofen and Musch, 2015). The VOC with a repeated measures correlation coefficient (**r_rm_**) |r_rm_| > 0.35 were identified using the National Institute of Standards and Technology NIST/EPA/NIH mass spectral library (NIST17; NIST, Gaithersburg, MD). The exhaled VOC identified at least at Level 2 were retained (Sumner et al., 2007) and are presented and referred to as the most discriminatory VOC.

Across the 3 lactation weeks of this study, the 32 healthy cows ingested, on average, 12.7 to 25.5 kg DM/d (mean: 20.9 kg DM/d), produced 20.9 to 50.1 kg milk/d (mean: 36.7 kg/d), and had serum BHB concentrations of 0.22 to 1.66 mmol/L (mean: 0.58 mmol/L), serum NEFA concentrations of 0.05 to 1.16 mmol/L (mean: 0.23 mmol/L), and a calculated EB of −84.1 to 52.6 MJ NEL (mean: −8.63 MJ NEL). During W5, W7, and W10, 100%, 67.7%, and 6.25% of the dairy cows exhibited NEB. For the 19 selected cows, concentrations of serum BHB (NEB: 0.29–1.66 mmol/L; PEB: 0.22–0.76 mmol/L; *P*-value_Wilcoxon test/PEB vs. NEB_ <0.01) and NEFA (NEB: 0.08 to 0.69 mmol/L; PEB: 0.05 to 0.44 mmol/L; *P*-value_Wilcoxon test/PEB vs. NEB_ <0.01) and exhaled VOC profiles (Q2 = 0.77, R2 = 0.84; Figure 1) differed between cows exhibiting NEB and PEB. Three discriminatory exhaled VOC were selected and identified as described previously: octanal, nonanal, and decanal. These 3 exhaled VOC are part of the chemical compound group of fatty aldehydes (Table 1). Higher concentrations of serum BHB and NEFA, as well as of exhaled octanal, nonanal, and decanal, were found for NEB cows compared with PEB cows (all *P*-value_Wilcoxon test/PEB vs. NEB_ ≤0.01; Table 1; Figure 2). The calculated EB was negatively correlated with serum BHB (r_rm_ = −0.62; $P_{r_{\mathrm{rm}}}$ < 0.01; Figure 2) and NEFA (r_rm_ = −0.48; $P_{r_{\mathrm{rm}}}$ < 0.01) concentrations and with exhaled octanal, nonanal, and decanal (r_rm_ = −0.40, −0.31, −0.53, respectively; all $P_{r_{\mathrm{rm}}}$ ≤ 0.01; Table 1; Figure 2). Serum BHB and NEFA concentrations were correlated with exhaled decanal (BHB: r_rm_ = 0.35; $P_{r_{\mathrm{rm}}}$ <0.01; NEFA: r_rm_ = 0.41; $P_{r_{\mathrm{rm}}}$ <0.01) but not octanal and nonanal (all |r_rm_| ≤ 0.14; all $P_{r_{\mathrm{rm}}}$ ≥ 0.30). The strength of the correlation between EB and serum BHB and NEFA did not differ from that of EB with octanal, nonanal, and decanal (*P* = 0.13, *P* = 0.09, and *P* = 0.22, respectively).

**Figure 1:**
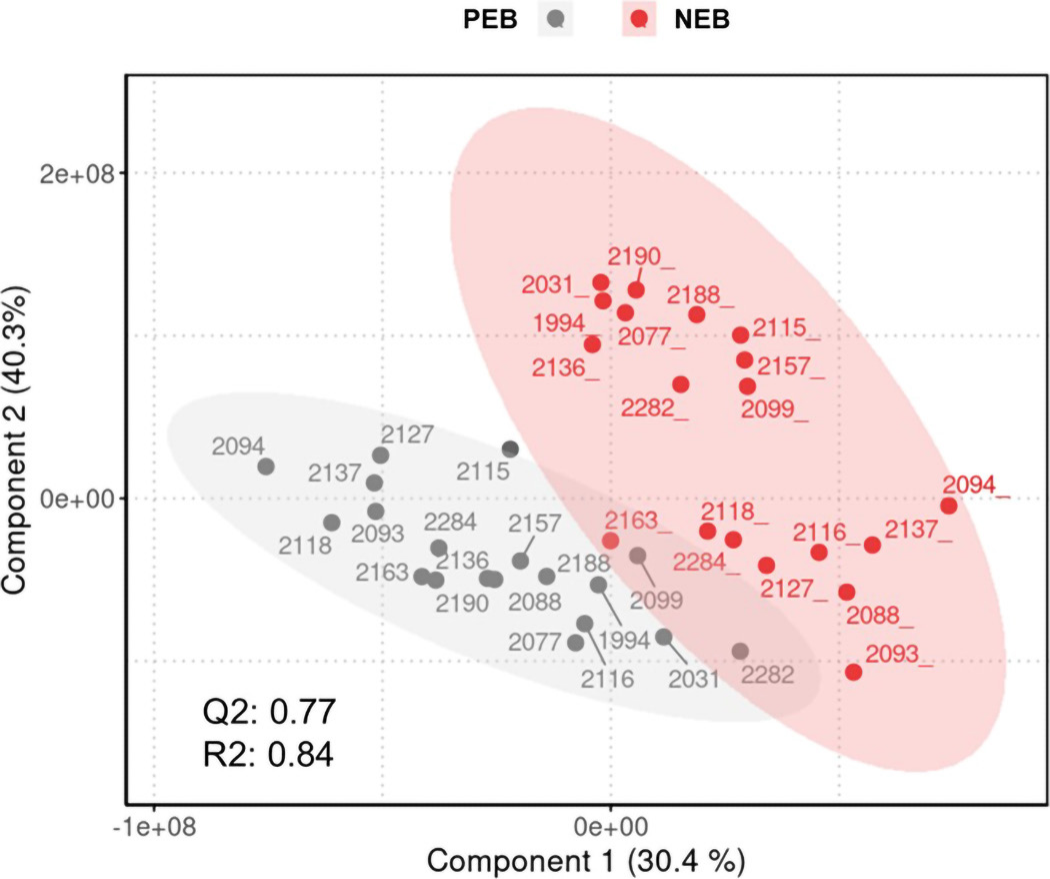
Partial least squares-discriminant analysis (PLS-DA) individual plots of components 1 and 2 presenting the discrimination of VOC profiles between NEB (red circles) and PEB (gray circles) states of dairy cows (n = 19). Circles are labeled by cow number. Q2 = predictive ability parameter; R2 = goodness-of-fit value of the PLS-DA model.

**Table 1:** Calculated EB, concentrations of serum BHB, serum NEFA, and relative concentrations of discriminatory VOC for cows (n = 19) with PEB and NEB

Item	PEB	NEB	SEM	*P*-value_Wilcoxon test/PEB vs. NEB_^1^	RT^2^	CAS^3^	Match factor (%)	RI calc^4^	RI ref^5^	Level^6^	TIC^7^ PEB	TIC NEB
EB (MJ NEL)	12.7	−32.2	4.51	<0.01								
BHB (mmol/L)	0.43	0.71	0.05	<0.01								
NEFA (mmol/L)	0.16	0.29	0.03	<0.01								
Exhaled VOC^8^												
Octanal			116,835	<0.01	14.96	124–13–0	83.7	1,001	1,003	2	1,737,127	2,348,897
Nonanal			200,703	0.01	16.80	124–19–6	83.2	1,098	1,102	2	2,030,067	2,574,259
Decanal			129,073	<0.01	18.44	112–31–2	82.1	1,200	1,206	2	2,584,915	3,422,639

1 *P*-value_Wilcoxon test/PEB vs. NEB_ = *P*-value calculated by the Wilcoxon's test comparing PEB and NEB states of cows after Benjamini–Hochberg's correction.

2 RT = retention time (min).

3 CAS = Chemical Abstracts Service registry number.

4 RI calc = calculated retention index using the temperature-programmed Kovats index (Girard, 1996).

5 RI ref = reference retention index of a nonpolar column 5 ms, ramp temperature (NIST17).

6 Level = identification level according to Sumner et al. (2007).

7 TIC = total ion count area.

8 VOC = volatile organic compound identified using National Institute of Standards and Technology NIST/EPA/NIH mass spectral library (NIST17; match factor > 80%) after manual peak integration using MassHunter Quantitative Analysis software (Agilent Technologies, Santa Clara, CA).

**Figure 2:**
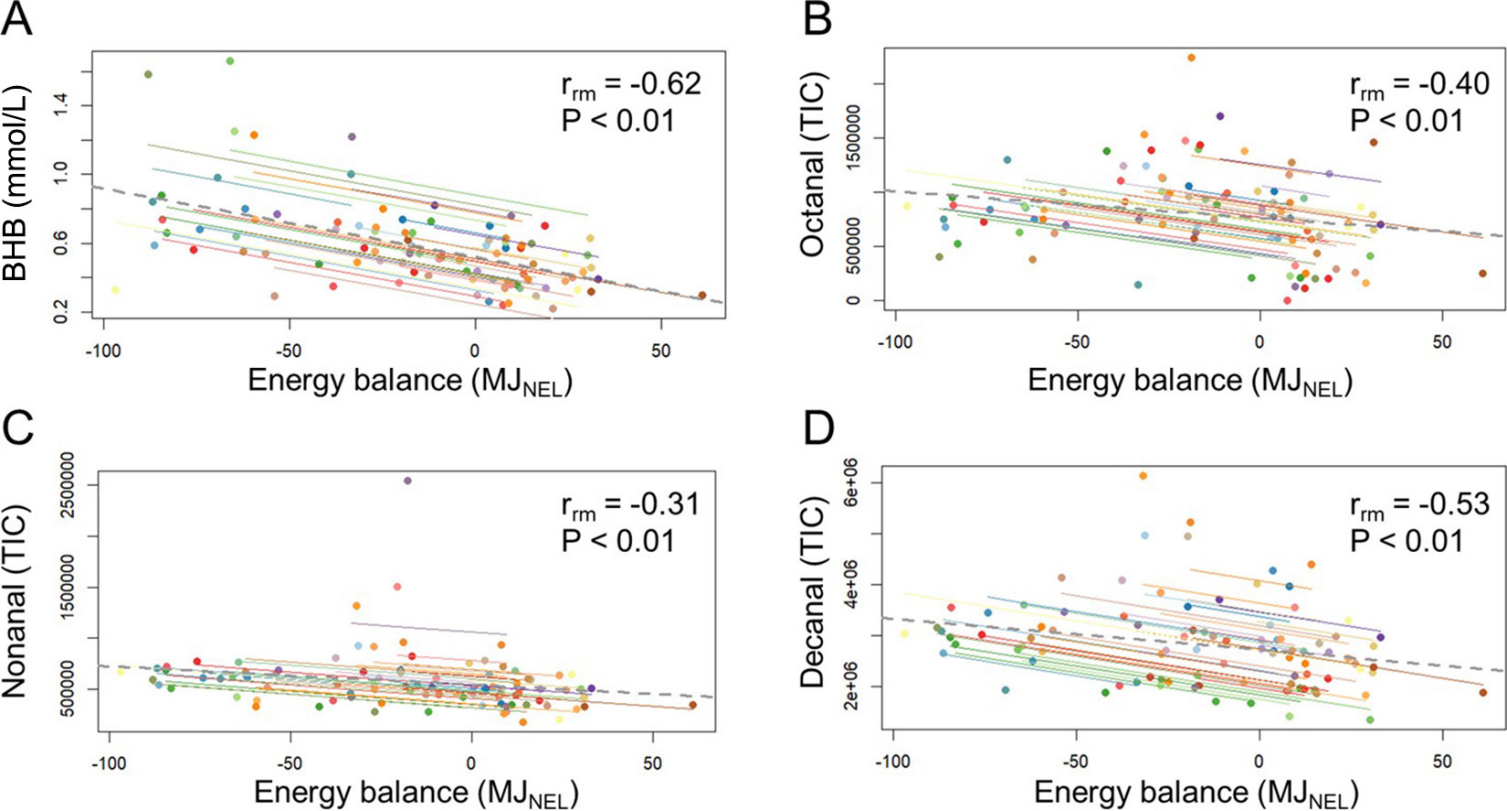
Plots of repeated measures correlations (r_rm_) between the calculated EB (MJ NEL) and (A) BHB (mmol/L), (B) exhaled octanal, (C) nonanal, and (D) decanal (total ion count [TIC]) using all 32 cows. Dots of the same color represent samples of 1 dairy cow across 3 lactation weeks; lines in the corresponding color show the linear regression line for the corresponding cow. Dotted gray line = overall regression line; r_rm_ = repeated measures correlation coefficient.

Our study employed an untargeted volatilomics approach to evaluate possible associations between exhaled VOC and EB in dairy cows. Furthermore, we assessed the strength of the correlations between EB and exhaled VOC compared with that of serum BHB and NEFA concentrations. In line with van Hoeij et al. (2019), the cows' serum BHB and NEFA concentrations were greater during the NEB states than during the PEB states. The differences might have been even more pronounced if sampling had occurred before W5. However, we deliberately excluded the immediate postpartum period, during which acute inflammatory processes (Huber, 2024) may confound the exhaled VOC profile. A NEB, together with reduced insulin sensitivity typical in early-lactating cows, enhances lipomobilization from adipose tissue, with the aim of meeting the energy demands of the animal (Ingvartsen and Andersen, 2000), but may also cause oxidative stress. Lipomobilization releases glycerol and NEFA; glycerol enters gluconeogenesis for glucose production—albeit this is often limited in early lactation. Nonesterified fatty acids have several possible fates: they can be oxidized via β-oxidation and further metabolization within the Krebs cycle to produce ATP, or—when β-oxidation is limited due to a lack of oxalacetate—they are instead converted into ketone bodies, such as BHB and acetone (van Erp-van der Kooij et al., 2023), which serve as alternative energy sources. When ketone body concentrations exceed certain thresholds, the animal is considered to be in ketosis. Another possible—but nonenergy-yielding—fate of NEFA is their partial oxidation into fatty aldehydes, for which this study provides some evidence.

The conversion from fatty acids into fatty aldehydes has been described earlier in conditions of oxidative stress, lipid breakdown, and within the fatty alcohol cycle in humans, for example, in lung cancer and skin fibroblasts (Rizzo, 2014; Sutaria et al., 2022). Both oxidative stress and lipomobilization are conditions that are also present in early-lactating cows during NEB (Song et al., 2021). Although not yet described for cattle, the fatty alcohol cycle most likely occurs in the liver of dairy cows, given its central role in mammalian lipid metabolism and the identification of involved enzymes, fatty acyl-CoA reductase, fatty alcohol dehydrogenase, and fatty aldehyde dehydrogenase, in the livers of various mammals, including cows (Lee et al., 1980; Tsutsumi et al., 1988). Therefore, we hypothesize that part of the NEFA released in NEB cows was metabolized into fatty aldehydes, released into the bloodstream, and, after crossing the blood–air barrier, exhaled. This mechanism has been described in humans, in whom exhaled fatty aldehydes serve as potential biomarkers for lung cancer and the associated oxidative stress (Sutaria et al., 2022). Oxidative stress might also help explain the chain length variability of the discriminatory exhaled fatty aldehydes identified in this study. Oxidative stress can shorten fatty acid chains in human adipose tissue and blood, and as a result, it can also shorten the chain length of their metabolization products, such as fatty aldehydes (Assies et al., 2014). Although NEFA released from adipose tissue have straight chains and may have been oxidized into the corresponding straight-chain aldehydes, oxidative stress processes may have contributed to producing the odd-chained aldehyde nonanal. The correlation between NEFA and the straight-chain fatty aldehyde decanal, but not nonanal, may support this hypothesis. The lack of correlation between octanal and nonanal with BHB and NEFA may suggest that their synthesis involves alternative pathways, for example, oxidative stress, rather than pathways directly linked to BHB and NEFA, as might be the case with decanal. However, the indicators of oxidative stress were not assessed in the present study.

The correlations between the calculated EB and serum BHB, exhaled octanal, nonanal, and decanal were all negative and moderate and did not differ in their strength. This finding supports our earlier argument. The positive correlations of decanal with NEFA align with previously reported positive correlations between serum NEFA and exhaled tetradecanal, another fatty aldehyde (Eichinger et al., 2025a). Serum BHB was negatively correlated with tetradecanal in that study, which is in contrast to the positive correlation between exhaled decanal and BHB and the absence of correlation of octanal and nonanal with BHB in the present study. The differences are potentially related to the later lactation stage and, therefore, to the less pronounced NEB and relatively low BHB concentrations and variability of the cows involved. An explanation for the differing associations between VOC and EB compared with those with NEFA and BHB may lie in the fact that NEB and elevated NEFA or BHB concentrations do not necessarily reflect the same metabolic state. Whereas NEB indicates an imbalance between energy intake and expenditure, elevated NEFA and BHB are markers of a catabolic state, which may accompany NEB but not in all cases. This is illustrated by the correlation plots (Figure 2), showing that serum BHB and exhaled fatty aldehydes varied considerably among cows with similar calculated EB. Such between-cow variation was described for BHB (Bruckmaier and Gross, 2017): a cow may be in NEB but still have low serum BHB concentrations. Concentrations of BHB are influenced by a wide range of physiological and environmental factors, including liver metabolic capacity, feed intake, stress, and individual adaptation strategies (Bruckmaier and Gross, 2017). This fact is used in breeding practices targeting reduced susceptibility to hyperketonemia and ketosis (VikingGenetics, 2021). These findings suggest that cows may differ in their metabolic pathways for coping with NEB, including how NEFA are processed to—ideally—meet energy demands. Possible consequences of this outcome for animal health remain to be investigated. Although the data analysis approach controlled for concentrate type, we must acknowledge potential confounders in the association between EB and exhaled fatty aldehydes. For example, the DMI was higher in W10 than in W5. Fatty aldehydes, including octanal, nonanal, and decanal, are present in fresh forage (Kigathi et al., 2009; Faulkner et al., 2018); thus, increased ingestion could lead to higher VOC exhalation, as reported earlier (Eichinger et al., 2025b). Additionally, the sampling time point may confound the relationship between EB and exhaled fatty aldehydes, given that cows had lower EB at the first sampling time point than at the third sampling time point and that the breath volatilome may change by the sampling time point (Eichinger et al., 2025b). Lactation stage and related physiological changes might also be confounders; for example, fatty aldehydes may play a role in thyroid metabolism and cell proliferation (reviewed by Rizzo, 2014). Further limitations of this study include the relatively narrow range of EB, BHB, and NEFA concentrations in our dataset, which may have limited the detection of stronger correlations. Therefore, the causality of the link between EB and fatty aldehydes that we found should be investigated in future studies without such confounders. Specifically, future research should identify the loci and underlying pathways of fatty aldehyde synthesis, examine fatty aldehyde transport mechanisms between organs and matrixes, and explore the role of oxidative stress in their formation. Such investigations should also include larger and distinct cohorts, as well as earlier lactation stages in which NEB is likely more pronounced. Investigating these factors will help determine whether, and, if so, which exhaled fatty aldehydes can serve as alternative biomarkers for NEB.
